# What Is the Role of Minimally Invasive Liver Surgery in Treating Patients with Hepatocellular Carcinoma on Cirrhosis?

**DOI:** 10.3390/cancers16050966

**Published:** 2024-02-28

**Authors:** Alessandro Vitale, Roberta Angelico, Bruno Sensi, Quirino Lai, Emanuele Kauffmann, Irene Scalera, Matteo Serenari, Michael Ginesini, Pierluigi Romano, Alessandro Furlanetto, Francesco D’Amico

**Affiliations:** 1 Department of Surgical Oncological and Gastroenterological Sciences, Padua University, 35122 Padua, Italy; alessandro.vitale@unipd.it (A.V.); romano.pierluigi5@gmail.com (P.R.); alessandro.furlanetto3@gmail.com (A.F.); 2 Transplant and HPB Unit, Department of Surgical Sciences, University of Rome Tor Vergata, 00133 Rome, Italy; 3 General Surgery and Organ Transplantation Unit, AUO Policlinico I of Rome, Sapienza University of Rome, 00185 Rome, Italy; quirino.lai@uniroma1.it; 4 Division of General and Transplant Surgery, Pisa University, 56126 Pisa, Italy; emanuele.kauffmann@unipi.it (E.K.); m.ginesini1@gmail.com (M.G.); 5 Unità di Chirurgia Epatobiliare e Trapianti di Fegato, Azienda Ospedaliero—Universitaria Consorziale Policlinico di Bari, 70124 Bari, Italy; irenescalera@gmail.com (I.S.); drdamico@hotmail.com (F.D.); 6 General Surgery and Transplant Unit, IRCCS Azienda Ospedaliero—Universitaria di Bologna, Sant’Orsola-Malpighi Hospital, 40138 Bologna, Italy; matteo.serenari@gmail.com; 7 Department of Medical and Surgical Sciences—DIMEC, Alma Mater Studiorum—University of Bologna, 40126 Bologna, Italy

**Keywords:** hepatocellular carcinoma, minimally invasive liver resection, laparoscopic ablation, liver transplantation, multi-parametric treatment hierarchy approach

## Abstract

**Simple Summary:**

HCC is a common tumor worldwide and a major cause of tumor-related mortality. Surgical treatments are available but often impractical in severely diseased patient with cirrhosis. In the past two decades, minimally invasive liver surgery has deeply changed the HCC treatment scenario. Today, it represents a superior treatment modality compared to open surgery due to the greater compliance of cirrhotic and obese patients, the possibility of laparoscopic ablation, and possibly better oncological results.

**Abstract:**

Minimally invasive liver surgery (MILS) has been slowly introduced in the past two decades and today represents a major weapon in the fight against HCC, for several reasons. This narrative review conveys the major emerging concepts in the field. The rise in metabolic-associated steatotic liver disease (MASLD)-related HCC means that patients with significant cardiovascular risk will benefit more profoundly from MILS. The advent of efficacious therapy is leading to conversion from non-resectable to resectable cases, and therefore more patients will be able to undergo MILS. In fact, resection outcomes with MILS are superior compared to open surgery both in the short and long term. Furthermore, indications to surgery may be further expanded by its use in Child B7 patients and by the use of laparoscopic ablation, a curative technique, instead of trans-arterial approaches in cases not amenable to radiofrequency. Therefore, in a promising new approach, multi-parametric treatment hierarchy, MILS is hierarchically superior to open surgery and comes second only to liver transplantation.

## 1. Introduction

Hepatocellular carcinoma (HCC) represents a major health issue worldwide as it is currently the seventh most common cancer (fifth among males and ninth among females) and the fourth cause of cancer-related death (sixth in females and second in males) [1]. Furthermore, while the highest incidence and mortality are registered in East Asian countries, its incidence is also markedly increasing in the Western world and it may soon become the third leading cause of cancer death [2,3].

HCC most often develops in the setting of chronic liver disease and especially in cirrhosis, the end-result of any chronic hepatic insult. The most commonly involved factors are hepatitis B virus (HBV), hepatitis C virus (HCV), alcohol, and metabolic-associated liver disease. They set in motion a series of events culminating in mutations in oncogenes or tumor-suppressor genes, which then promote cancer development through specific pathways (see next section).

Thus, patients with HCC often present with various clinical complexities due to the underlying liver conditions and the patient’s comorbidities which inevitably affect management [4]. In particular, the substrate of liver cirrhosis substantially limits the applicability of many of the currently used therapeutic modalities, thus significantly magnifying the complexity of treatment. In fact, given the vital role of the liver in human survival, all treatments must take into consideration post-procedural residual liver function. For example, surgical resection must leave untouched at least 25% of “normal” liver and up to 40% of cirrhotic parenchyma. This means that resection is not considerable in cases with extensive tumor burden. Furthermore, patients with severe cirrhosis (classified as Child–Pugh B or C) may not be able to tolerate resection independently from its volume, as they are poorly compliant with any perturbation to their fragile homeostasis.

Moreover, patients with metabolic-associated steatotic liver disease (MASLD) frequently suffer from multiple metabolic-related comorbidities such as ischemic heart disease, diabetes, and reduced respiratory reserve and are at higher risk of postoperative complications.

In this context, the minimization of therapeutic “invasiveness” (i.e., the impact on a patient’s delicate homeostasis) could be viewed as being of paramount importance.

Laparoscopic surgery was introduced three decades ago and has since been demonstrated to offer superior results compared to open surgery for most indications. In the setting of liver disease, its establishment has been slower due to its intrinsic difficulties, including vascular hazards, anatomical position, etc., but today it represents not only a major alternative to open surgery but probably the preferred option (Section 10).

In fact, minimally invasive liver surgery (MILS), such as laparoscopic and robotic techniques as well as image-guided ablation therapies, have emerged and established themselves as effective alternatives to traditional open surgery for patients with HCC [5,6]. As for other cancers, minimally invasive surgery has brought relevant advantages regarding early postoperative outcomes [7,8]. However, whether the minimally invasive approach determines further benefits in terms of expanding the HCC surgical indications, modifying the HCC treatment algorithm and long-term oncological outcomes, is still debated.

**Figure 1:**
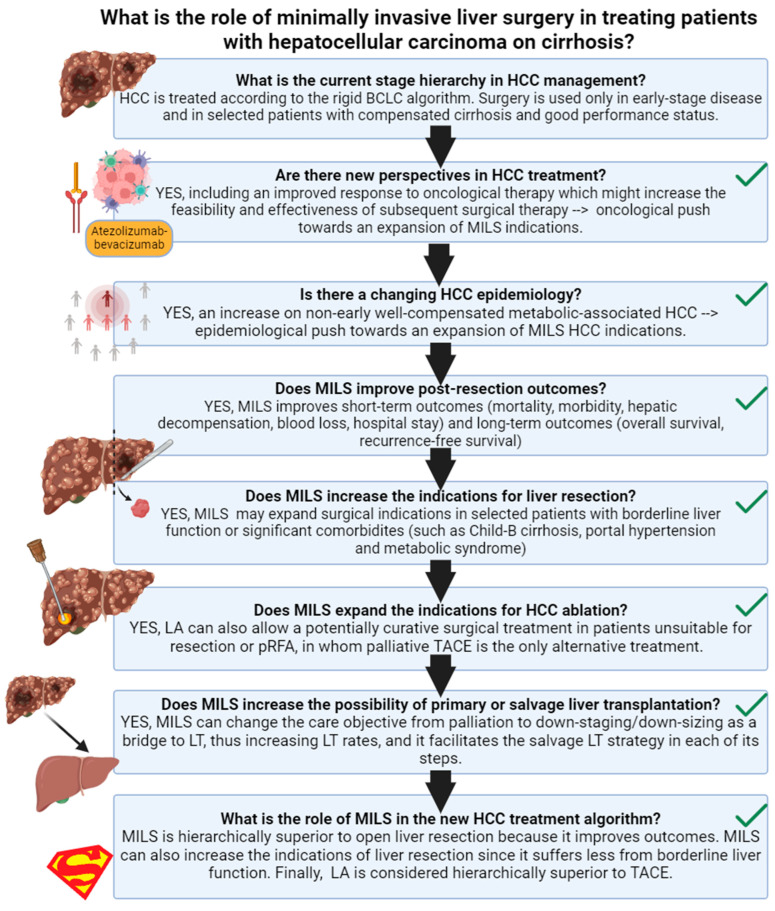
Review outline. Created in BioRender. HCC: hepatocellular carcinoma; MILS: minimally invasive liver surgery; LA: laparoscopic ablation; BCLC: barcelona clinic liver cancer; TACE: trans-arterial chemo-embolization.

This narrative review aims to deeply analyze the current role of the minimally invasive liver surgical approach in treating patients with HCC on cirrhosis and its future perspectives (Figure 1).

## 2. Natural History of HCC

HCC has a complex pathophysiology guided by an interplay of factors including genetics, cellular microenvironment, immune cells, and interaction with risk factors such as alcohol or viruses. This translates into an intricate clinical pattern in which the history of the tumor and of the underlying predisposing disease are tightly interwoven. Next-generation sequencing has identified many oncogenic or tumor-suppressive genes which are recurrently mutated in HCC. Viral insertions, chromosome translocation, the amplification of certain genes, and the activation of telomerase are altered in 80% of HCC cases. Most commonly involved pathways are the Wnt–β-catenin (CTNNB1, AXIN1, APC), mTOR, MAPK cell cycle control (RB1, TP53, CCNE1, CCNA2, ARID1A, ARID2, PTEN, RPS6KA3, NFE2L2), oxidative stress, and epigenetic regulation [9]. As with many other cancers, in the great majority of cases, the genesis of HCC is related to chronic insults to hepatocytes, which induce concomitant liver disease and very frequently full blown cirrhosis. Thus, the predisposing factors involved are the same as those of the underlying liver affliction, such as hepatotropic viruses, alcohol, dysmetabolism, hemochromatosis, and Wilson’s disease. In fact, as we shall see, liver conditions are as important as the disease stage in directing therapeutic strategies and possibilities. Symptoms of HCC are generally absent and patients tend to manifest those related to the underlying cirrhosis including ascites and variceal hemorrhage. For this reason, in the absence of close surveillance, HCC tends to be diagnosed at an advanced stage and has historically carried very poor survival (12% five-year overall survival) [10]. On the contrary, early-stage disease tends to be relatively indolent, especially when a single nodule is present, and represents fertile ground for potentially curative treatment. Surveillance in cirrhotic patients is therefore both of paramount importance for liver disease patients’ health and cost-effective.

## 3. Current Management of HCC

The management of HCC remains quite unique in the oncological panorama in that it is inextricably bound and limited by the severity of liver involvement. Most specialized centers have so far based their clinical activity on the suggestions included in the BCLC (Barcelona Clinic Liver Cancer) algorithm [11]. This algorithm essentially represents a stage-based approach. In fact, it focuses on the correct staging of the disease, followed by an evaluation of liver function and performance status, and the consequent attribution to a specified treatment modality. According to BCLC, for early-stage disease, curative therapies include radiological ablation, surgical resection, and liver transplantation. All these methods have their precise indications, and their relevant limitations. For single lesions, <3 cm in diameter, radiological ablation can be considered a curative technique, while its performance is significantly limited for larger nodules. Surgical resection is more appropriate in larger nodules or multinodular disease, as it demonstrates superior oncological outcomes, yet it necessitates a sufficiently large liver remnant to avoid the risk of post-hepatectomy liver failure, a considerable limitation when considering that at least 40% of parenchyma should be spared when dealing with cirrhotic livers. Liver transplantation is known to offer the best possible long-term results as it eliminates both the current disease (HCC) and its substrate (the cirrhotic liver), decreasing the risk of post-surgical recurrence. However, the procedure is intrinsically high-risk and, more importantly, it suffers from organ shortage, and thus availability is restricted to patients who satisfy certain internationally accepted criteria. Loco-regional therapy such as trans-arterial chemo-embolization (TACE) or trans-arterial radio-embolization (TARE) are essentially considered palliative procedures to be used in intermediate stages, as evidence of the possibility of achieving cure with these techniques is currently lacking. Nonetheless, they are very useful in the context of treating patients whose disease falls outside transplant criteria, as they are often also used as complementary measures for down-staging, eventually facilitating the path to liver transplantation. Finally, systemic therapy is reserved for advanced stages and best supportive therapy for those patients whose performance status or hepatic reserve is so compromised that they are not able to tolerate systemic therapy.

While the BCLC algorithm remains an important guidance, its intrinsic rigidity has been identified as one of the main shortcomings. Despite attempts at introducing increased clinical liberty in the selection of treatment, such as the concept of “stage migration”, the system appears to remain an under-representation of contemporary treatment armamentarium. We shall see in the next paragraphs how perspectives and treatment modalities are rapidly evolving in the field.

## 4. Are There New Perspectives in HCC Treatment?

In recent decades, we have been observing the progressive evolution of surgical and medical treatments directed at HCC management. The major novelty has been the introduction of effective systemic therapies, particularly immunotherapy. These therapies are increasing the conversion rate from non-resectable tumors to resectable tumors [12]. Most of the available data concern the use of atezolizumab, a humanized anti-PD-L1 antibody. In one study, the atezolizumab/bevacizumab combination converted 8.6% of patients from unresectable to resectable HCC [12]. Moreover, combining systemic and loco-regional therapy may considerably further increase this rate [13]. A recent meta-analysis showed that the association between trans-arterial chemo-embolization (TACE), tyrosine-kinase inhibitors (TKIs), and immune checkpoint inhibitors (ICIs) has demonstrated the ability to convert 42% of patients from inoperable to operable disease [14]. Another effective regimen might be TACE followed by hepatic arterial infusion chemotherapy, as reported by Li et al. [15]. Moreover, in patients who successfully underwent conversion therapy, the subsequent surgical treatment appears to be associated with more prolonged survival compared to non-surgical management, albeit with possibly increased perioperative risk [16,17,18,19]. Finally, the new IMBRAVE trial has shown the effectiveness of adjuvant therapy for the first time in decreasing the risk of post-surgical tumor recurrence in high-risk HCC patients, which was confirmed in widespread reproducibility studies [20,21,22,23,24].

Thus, the answer to the first question is YES, there are new perspectives in HCC treatment, including an improved response to oncological therapy which might increase the feasibility and effectiveness of subsequent surgical therapy. This may result in an oncological push towards the expansion of MILS indications.

## 5. Is There a Changing Scenario in the Epidemiology of HCC?

There has been an ongoing epidemiological transition of the underlying liver-related disease leading to HCC occurrence, which might have relevant implications for HCC management.

The incidence of HCC is widely known to be correlated to the process of cirrhosis in livers with predisposing risk factors. The most commonly involved determinants are HBV, HCV, alcohol abuse, and MASLD. Worldwide, the most common etiologic factors are HBV and HCV but, especially in the Western world, this is rapidly changing due to effective anti-viral therapy and the “metabolic pandemic” that we are currently facing [9].

Nowadays, we are observing a steady decline in virus-related HCC with a concomitant rise in metabolic-associated HCC [25]. The incidence of metabolic-related HCC is increasing worldwide and currently represents the fastest growing cause of HCC, with similar reports from Europe, North America, and East Asia [25,26,27,28,29]. Patients with MASLD, especially in the presence of diabetes, may be at particular risk [30]. In Italy, this epidemiological trend has been particularly pronounced, as recently shown by Vitale A et al. [31], who demonstrated that metabolic-associated HCC is overtaking HCV-related HCC. Metabolic-associated HCC is generally more advanced, multinodular, and more often diagnosed in advanced stage than virus-related HCC [31,32,33]. Nonetheless, metabolic-associated HCC may be biologically less aggressive in terms of cancer-related death risk [31], as emerging from surgical series [34,35,36]. More importantly, metabolic-associated HCC differs from other etiologies because it might arise in a non-cirrhotic liver in as many as 20–38.5% of cases [36,37], and which patients require strict surveillance remains unclear [32]. In an effort to identify them, novel markers are being considered for HCC screening. The protein-induced by vitamin K absence (PIVKA) represents a new tool for HCC diagnosis as it carries better sensitivity than AFP [35]. Nevertheless, it still does not fulfill the criteria to be used routinely in the clinical setting, and it is suggested to be tested along with AFP. Other markers as the lens culinaris-agglutinin reactive (AFP-L3), inflammatory markers, the overexpression of oncogenes, or the downregulation of oncosuppressors and microRNA are emerging approaches, but there is still no consensus for the cut-off to be used for each marker and no competing risk analysis of them either [38]. Ongoing studies aim mainly to find a correlation between marker and early disease, to better fight the tumor. Liquid biopsy includes the analysis of circulating HCC cells and aims to perform a non-invasive sampling to predict prognosis, stage, and response to therapy, but its use in the clinical setting needs to be validated [39]. In general, the emergence of MASLD-related HCC translates into a need for revising surgical strategies. More and more HCC patients will be, on one hand, able to better tolerate more extensive hepatic resection, but on the other hand, more prone to cardiovascular morbidity. In this context, a more extensive use of the minimally invasive surgical approach for major hepatic resection or anatomical hepatic resection could thus be foreseen, aiming to minimize the physiological impact of the surgical procedure.

Therefore, the answer to the second question is YES. We are going through a new HCC epidemiology: an increase in non-early well-compensated metabolic-associated HCC. This translates in an epidemiological push toward an expansion of MILS HCC indications.

## 6. Does MILS Improve Post-Resection Outcomes?

The question of whether minimally invasive liver surgery (MILS) improves post-resection outcomes for HCC is a topic that has garnered considerable attention in the literature. Numerous studies provide substantial evidence supporting the significant advantages of MILS in enhancing short-term surgical outcomes for patients undergoing HCC resection when compared to the traditional open technique, particularly in high-volume centers [40,41,42,43,44,45]. Both laparoscopic and robotic techniques have demonstrated their efficacy in reducing the risk of post-hepatectomy liver failure and mitigating postoperative complications [46,47].

Nevertheless, the discussion surrounding the influence of MILS on long-term outcomes post HCC resection is characterized by nuanced debates, primarily resulting from the dearth of randomized trials and conflicting evidence among available studies. While a majority of studies report comparable outcomes between MILS and the open technique, emerging evidence suggests that the benefits of MILS may extend beyond the short term to positively impact long-term oncological endpoints [48,49]. A recent large cohort study with propensity score matching from Japan and Korea showed that laparoscopic liver resection had a better mid- and long-term disease-free survival than open hepatectomies [50]. Another multicenter study showcased a significant trend in robotic liver resection, indicating improved disease-free survival and overall survival when compared to open surgery [44]. These data have been confirmed by several updated meta-analyses [51,52,53,54,55], which conclude that MILS improves short-term and long-term oncological outcomes.

Despite the robust association between minimally invasive surgery and improved long-term outcomes, the underlying mechanisms driving this phenomenon remain elusive. One prevailing hypothesis attributes most of the benefits to the marked reduction in the risk of post-hepatectomy liver failure. Additionally, the oncological outcomes of MILS may be further optimized by conducting more frequent anatomic liver resections when feasible. This is substantiated by recent randomized clinical trials [48,56] and the escalating utilization of innovative techniques, such as indocyanine green fluorescence navigation. This navigational approach has exhibited promise in enhancing the achievement of liver resections with tumor-free margins, as evidenced by a published study indicating increased disease-free survival after MILS with indocyanine green fluorescence navigation compared to MILS without it [57]. Moreover, the expeditious recovery observed in MILS patients facilitates a quicker return to adjuvant therapy [58].

These comprehensive findings strongly suggest that MILS holds the potential not only to improve mid-term outcomes but also to exert a positive influence on long-term outcomes following liver resection. The anticipation is that further advancements will be witnessed with the widespread adoption of new technologies. Table 1 provides a meticulous summary of key studies reporting HCC oncological outcomes treated with minimally invasive surgical techniques [46,48,49,50,51,52,53,54,55].

Therefore, the answer to the third question is YES.

## 7. Does MILS Increase the Indications for Liver Resection?

In recent decades, an expansion of indications for liver resection in patients with HCC has been observed due to the refinements of the surgical technique [59]. In this context, several studies reported that MILS represents a favorable prognostic factor for achieving complete tumor resection (R0) [60,61]. In fact, MILS appears to be inversely correlated to the risk of postoperative liver failure and to increase the probability of achieving textbook outcomes which, in turn, improves overall survival [60,61].

Few studies have addressed specific HCC-affected populations such as cirrhotic or MASLD patients. Liver resection by minimally invasive technique was shown to decrease major morbidity and to prolong survival in patients with HCC and Child-B cirrhosis, and to reduce major postoperative complications and liver failure rates in patients with comorbid metabolic syndrome [62,63]. In both these populations, MILS was recognized as a favorable prognostic factor, although patients with Child B-7 cirrhosis may benefit more than those with Child B-8/9 cirrhosis [63,64,65,66].

Metabolic syndrome is a complex clinical entity, including hypertension, dyslipidemia, obesity, and insulin resistance. MASLD has a variable impact on liver function but is directly associated with carcinogenesis, with a significantly higher risk of developing HCC. MASLD patients have an augmented risk of morbidity and mortality after liver surgery, based on the severity of parenchymal injury and on the coexistent metabolic, vascular, and cardiological comorbidities, which strongly impact outcome. In these patients, a particular effort in mitigating the impact of complications on the postoperative course is warranted. However, despite potentially benefiting from a minimally invasive approach, obesity and comorbidities can frequently interfere with a robotic or laparoscopic approach on a technical point of view [62]. A similar concept may be proposed for elderly patients. There is wide consensus on the fact that anagraphical age is not a sufficient parameter to exclude otherwise fit patients from treatment. However, the incidence of severe comorbidities in elderly patients is significantly higher, and a thorough multi-parametric analysis of the biological age and fitness of the patient is mandatory.

From this perspective, the minimally invasive approach may compensate other adverse prognostic factors for the surgical treatment (such as Child-B cirrhosis, portal hypertension, and metabolic syndrome), and, consequently, it might expand the surgical indications in selected patients with borderline liver function or significant comorbidities [67]. Table 2 summarizes the main studies identifying MILS as a favorable prognostic factor [60,61,63,64].

## 8. Does MILS Expand the Indications for HCC Ablation?

The laparoscopic ablation (LA) of HCC represents another minimally invasive surgical technique that may increase the indications for surgery [68]. LA can be used when patients are unsuitable for both formal liver resection and percutaneous radiofrequency ablation (pRFA). LA permits HCC ablation without imparting an excessive insult to the hepatic parenchyma, and can provide a potentially curative alternative to TACE, which instead represents a palliative procedure. However, the lack of strong evidence on ample populations, along with technical and logistic unavailability, still strongly limits the adoption of this technique.

The laparoscopic approach to microwave ablation allows several technical issues to be overcome. The first objective advantage is the possibility to have a freer access to the liver, with several operative angles both for the ultrasound probe and for the needle. In addition, abdominal organs that might be interposed between the abdominal wall and the liver can be generally easily displaced. A second major advantage of a laparoscopic approach is the direct and effective control of any occurring bleeding by means of coagulative or surgical means. This is of paramount importance in the presence of cirrhosis-related coagulopathy, featuring both low platelet count and coagulation factor imbalance.

Recently, an Italian study reported that when textbook outcomes are reached, LA may indeed prove to be a curative therapy [69]. The same study group conducted a large multicenter study (as yet unpublished), comparing three cohorts of patients undergoing pRFA, laparoscopic microwave ablation (LA), or TACE. After weighing and balancing for heterogeneities of the three cohorts, we observed that the outcome of LA is similar to that of pRFA and superior to that of TACE.

For small nodules, even in patients with a high risk of decompensation, LA allows a radical, potentially curative approach without the incumbent risks of surgical resection.

Therefore, MILS has the potential role of increasing the span of indications to surgery as it can compensate for other adverse prognostic factors, allowing surgery in patients at higher risk of postoperative liver failure or major morbidity (i.e., cirrhosis-related portal hypertension, Child-B cirrhosis, MELD > 10, or metabolic syndrome). Moreover, LA can also allow a potentially curative surgical treatment in patients unsuitable for resection or pRFA, in whom palliative TACE is the only alternative treatment.

Therefore, the answer to this fourth question is YES.

## 9. Does MILS Increase the Probability of Primary or Salvage Liver Transplantation?

In selected patients with cirrhosis, liver transplantation (LT) represents the gold standard treatment for non-resectable HCC tumors [23]. The HCC selecting criteria for LT are constantly expanding [70,71], and nowadays, HCC represents the leading indication for LT [72,73]. In this scenario, MILS emerges as a transformative factor that could impact access to LT for HCC in two distinct scenarios: primary LT and salvage LT. In the first scenario, patients who are typically beyond the criteria for translatability, deemed unfit for formal resection, and with tumors in locations making radiofrequency ablation (RFA) unfeasible, find a potential solution in a minimally invasive approach with loco-regional ablation (LA). This proves to be an effective down-staging/downsizing strategy for HCC, potentially paving the way for subsequent LT. The Padua Liver Transplantation Centre’s experience attests to the success of this aggressive approach, achieving down-staging in an impressive 88% of HCC patients—a proportion significantly surpassing that observed in the XXL study, which relied solely on radiological locoregional treatments [71,74].

In the second scenario, patients who undergo primary HCC resection with an intention-to-treat curative-intent resective strategy but develop tumor recurrence can be treated by salvage transplantation if the tumor characteristics are within transplant criteria [75]. Regarding salvage LT outcomes, patients approached by MILS for the initial tumor resection seem to have benefits compared to open approaches. According to a multicenter national retrospective study conducted by Levi Sandri et al., MILS was a protective factor against the risk of LT delisting, post-transplant death, and HCC recurrence [76]. Several studies demonstrated that the beneficial effects of MILS on salvage LT outcomes may be due to lower adhesion and to easier and less hemorrhagic transplantation surgery in patients who received a previous minimally invasive liver resection compared to those treated with the open technique [76,77,78]. Moreover, a recent study has shown that the probability of receiving salvage LT is higher when resection is performed in a center with a liver transplant program [79]. All these data suggest that MILS can change the care objective from palliation to down-staging/down-sizing as a bridge to LT, thus increasing LT rates. Moreover, it facilitates the salvage LT strategy in each of its steps. A summary of the studies analyzing the effects of MILS on LT is reported in Table 3 [76,77,78]. Therefore, the answer to this fifth question is YES.

**Figure 2:**
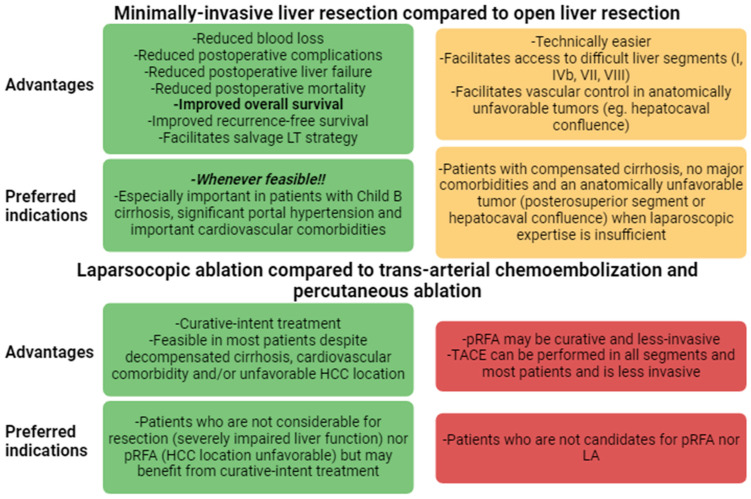
Advantages of MILS over open surgery and TACE/RFA and preferred indications. HCC: hepatocellular carcinoma; LA: laparoscopic ablation; pRFA: percutaneous radiofrequency ablation; TACE: trans-arterial chemo-embolization.

Figure 2 summarizes the overall advantages and preferred indications of MILS.

## 10. What Is the Role/Position of MILS in the HCC Treatment Algorithm?

In 2022, the latest version of the Barcelona Clinic Liver Cancer (BCLC) algorithm, also endorsed by the American Society for the Study of Liver Disease (AASLD), was released [11,23]. From the updated BCLC recommendations, there was an expansion of HCC indications for LT, while no advances were assigned to liver resection in the treatment algorithm. In particular, MILS has no specific role in patients with early HCC and borderline liver function, in patients with oligo-nodular or intermediate HCC (Stage B), or in patients with intrahepatic venous invasion.

Conversely, the answer to the initial question is deeply developed in a novel framework proposed in a recent policy review on HCC treatment allocation [80]. This framework has also been reproduced in Figure 1.

As discussed in a *Lancet Oncology* paper [80], the central limit of the BCLC system is its intrinsic “stage hierarchy” nature. Stage hierarchy means that HCC stages or sub-stages dictate treatment choice, increasing the patient’s risk of undertreatment. An exciting alternative to the stage hierarchy philosophy is the ordinal therapeutic hierarchy approach, in which treatment choice is totally or partially independent from the tumor stage, and treatment options are hierarchically ordered according to survival benefits [81,82]. However, an utterly liberal treatment hierarchy approach may increase the patient’s risk of over-treatment [81]. A solution to the limits of both stage and therapeutic hierarchy concepts is the recent proposal of a multi-parametric treatment hierarchy approach (Figure 1) [80]. In this proposal, the multi-parametric evaluation of a multidisciplinary expert team balances the ordinal therapeutic hierarchy risk of overtreating HCC patients [65].

**Figure 3:**
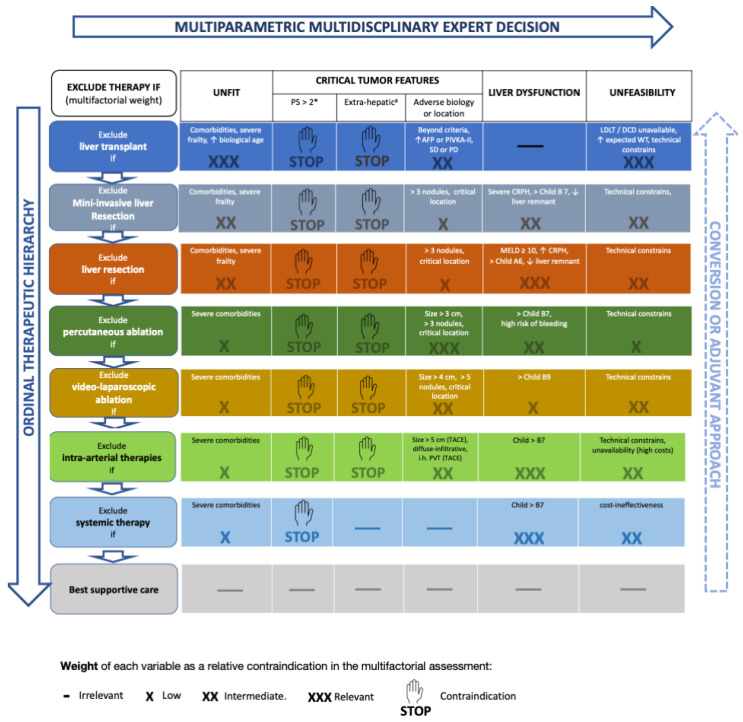
The novel framework includes the concepts of multi-parametric and converse therapeutic hierarchy. This figure is derived from Ref. [68]. # Performance status expresses tumor-related symptoms and, therefore, tumor aggressiveness. * Extrahepatic metastases, invasion of the main trunk of the portal vein or inferior vena cava. The right-side arrow indicates the concept of “converse therapeutic hierarchy” (conversion or adjuvant approach). The arrow is dashed and faded since the evidence supporting this concept is still weak. Abbreviations: PS, performance status; AFP, alpha-fetoprotein; PIVKA-II, Protein Induced by Vitamin-K Absence-II; LDLT, living donor liver transplantation; DCD, donor after circulatory death; MELD, model for end-stage liver disease; CRPH, clinically relevant portal hypertension; TACE, trans-arterial chemo-embolization; PVT, portal vein thrombosis. Up arrow: increased/excessive/advanced; Down arrow: insufficient.

As can be seen in the novel framework (Figure 3), MILS is hierarchically superior to open liver resection because it improves mid-term outcomes (Table 1).

Moreover, MILS can also increase the indications of liver resection since it suffers less from borderline liver function (Table 2). This aspect is also graphically underlined in the novel framework (Figure 3), where liver dysfunction has a higher probability to contraindicate open (three crosses) than minimally invasive liver resection (two crosses).

Finally, tumor ablation using LA is considered hierarchically superior to TACE, so before deciding to use intra-arterial therapy, the feasibility of LA should be considered [68,69]. This novel operative framework is fashioned to assist the decision-making of experts in the setting of a multidisciplinary meeting. The role of new systemic therapies is under scrutiny at present. However, if the auspices of a potential down-staging effect were confirmed, the scenario would become additionally complex. This “conversion-to-surgery” potential is made clear by the concept of “converse therapeutic hierarchy”, meaning that in case of a successful down-staging, previously discarded therapeutic options should be again taken into consideration, in order to offer the best possible treatment to the patient at any given time. The interesting feature of this novel operative framework relies on it being an easily adaptable tool that can be fashioned on the needs of the specific patients. 

## 11. Conclusions

The HCC panorama is in continuous evolution. The etiology of the underlying liver disease is changing from virus- to metabolic-associated. The scientific community is devoting ample resources to developing novel therapeutic options, both in terms of systemic therapy and surgical techniques.

In this complex setting, minimally invasive liver surgery is a strong tool that has the potential to broaden the access to curative treatments (resection or ablation) to several patients, by reducing the surgical trauma and consequent impact on morbidity and mortality.

In particular, MILS offers superior short-term results compared to open surgery and appears to improve long-term survival. While more and more patients will be oncologically candidable for resection due to the reduction in tumor burden brought about by an improved response to systemic therapy, MILS will effectively expand the indications as it increases compliance to surgery, a factor of major importance especially in the setting of Child B-7 and MAFLD patients. Laparoscopic ablation should also be taken into highest consideration as a potentially curative option, hierarchically superior to TACE. Moreover, minimally invasive techniques, by having low impact and being potentially repeatable, effectively serve as down-staging procedures as a bridge to liver transplantation, and in this context, laparoscopic ablation may be superior to radiological techniques. In the context of salvage LT, MILS can decrease rates of delisting, postoperative mortality, and HCC recurrence. Overall, MILS has been established as the most important surgical tool in HCC management, second only to liver transplantation, and it must thus be considered hierarchically superior to alternative techniques such as open surgery and radiological approaches.

The management of this changing and intricate world requires an expert multidisciplinary approach and a continuous effort to interpret the wide spectrum of interplaying factors that concur in every patient reality. This personalized medicine approach reflects the need to offer the right treatment for the right patient at the right time.

## Figures and Tables

**Table 1:** Key studies comparing outcomes of MILS vs open surgery for HCC.

Year	Author	Journal	Study Type	N° of Patients	Outcomes
2016	Tan To Cheung et al. [49]	*Annals of Surgery*	Retrospective Propensity score matching	110 MILS 330 OPEN	-Increased 1-, 3-, and 5-year overall survival (98.9%, 89.8%, and 83.7% in the laparoscopic group, and 94%, 79.3%, and 67.4% vin the open group)
2018	Goh et al. [51]	*International Journal of Surgery*	Meta-analysis (five non-randomized case-matched studies)	276 MILS 612 OPEN	-Lower tumor recurrence -Increased 1-, 3-, and 5-year overall survival -Increased disease-free survival at 1 year
2019	Jiang et al. [52]	*Journal of Lap and Advanced Surgical Techniques*	Meta-analysis (17 non-randomized case-matched studies)	798 MILS 1206 OPEN	-Increased 1-, 3-, and 5-year overall survival -Increased disease-free survival at 1 year -Decreased blood loss and transfusion rates -Decreased postoperative complications -Wider surgical margin -Decreased postoperative hospital stay -Decreased mortality
2021	Pan et al. [55]	*Frontiers in Oncology*	Meta-analysis (12 non-randomized case-matched studies)	784 MILS 1191 OPEN	-Increased 1-, 3-, and 5-year overall survival -Decreased overall and major complications -Decreased porstoperative mortality -Decreased postoperative liver failure and ascites
2021	Kabir et al. [53]	*British Journal of Surgery*	Meta-analysis (11 non-randomized case-matched studies)	690 MILS 928 OPEN	-A 16% reduction in the hazard ratio of death -Decreased length of stay -Decreased blood loss -Decreased overall and major complications
2022	Kaibori et al. [50]	*Liver Cancer*	Retrospective Propensity score analysis	146 MILS 807 OPEN	-Increased overall disease-free survival -Increased disease-free and overall survival for sub-group of patients with single HCC or >4 cm. -Decreased postoperative complictions -Decreased postoperative hepatic decompensation
2022	Kamarajah et al. [54]	*Scandinavian Journal of Surgery*	Meta-analysis (50 non-randomized studies)	4071 MILS 9660 OPEN	-Increased 3-year disease-specific mortality -Increased 5-year all-cause mortality
2023	Di Benedetto et al. [46]	*JAMA Surg*	Retrospective Propensity score matching	106 MILS (robotic) 106 OPEN	-Decreased post-hepatectomy liver failure -Decreased hospital stay -Decreased admissions to intensive care
2023	Kato et al. [48]	*Cancers*	Retrospective Propensity score matching	91 MILS 91 OPEN	-Longer overall survival and recurrence-free survival -Decreased blood loss and transfusion rates -Decreased 90-day morbidity and mortality -Decreased bile leak -Decreased postoperative hospital stay

**Table 2:** Main studies identifying MILS as a favorable prognostic factor.

Year	Author	Journal	Study Type	N° of Patients	Results
**2019**	Prodeau et al. [61]	*Journal of Hepatology*	Prospective observational study	112 MILS 231 OPEN	MILS was independently inversely correlated to the likelihood of postoperative liver failure.
**2020**	Hobeika et al. [60]	*JHEP Reports*	Prospective observational study	267 MILS 158 OPEN	MILS was independently correlated to textbook outcomes.
**2020**	Berardi et al. [63]	*Journal of Hepatology*	Retrospective study	122 MILS 131 OPEN	MILS was independently inversely correlated to major morbidity in Child-B cirrhosis.
**2023**	Berardi et al. [64]	*Hepatology*	Retrospective study	445 MILS 642 OPEN	MILS was independently inversely correlated to major morbidity in patients with metabolic syndrome.

**Table 3:** Main studies on the effect of MILS on LT.

Year	Author	Journal	Study Type	N° of Patients	Outcomes
**2009**	Laurent et al. [77]	*Journal of Hepatobiliary and Pancreatic Surgery*	Retrospective monocentric study	12 MILS 12 OPEN	LT after MILS was correlated to reduced operative time, blood loss, and transfusion requirements.
**2018**	Rhu et al. [78]	*Annals of Surgical Treatment and Research*	Retrospective monocentric study	10 MILS 52 OPEN	MILS reduced intra-abdominal adhesions during salvage LT.
**2020**	Levi Sandri et al. [76]	*Liver Transplantation*	Retrospective multicentric study	44 MILS 167 OPEN	MILS was independently correlated to a reduced risk of delisting, post-transplant death, and HCC recurrence.
